# Observations on the Religious Delusions of Insane Persons, &c.

**Published:** 1842-04-01

**Authors:** 


### Observations on the Religious Delusions of Insane Persons; and on the Practicability, Safety, and Expediency of imparting to them Christian Instruction, &c. &c. &c. By *Nathaniel Bingham*, M. R. C. Surgeons, &c. Octavo, pp. 213. Hatchard, Piccadilly, 1840.

It is not our intention to analyse this work, which we did not see till very recently, though published nearly two years ago. It is more popular than professional, though written with good sense and a fair amount of information on the ticklish subject of insanity. To the "*religious* delusions of the insane" we attach no more importance than to any of their other delusions; nor are we at all convinced by Mr. Bingham that, *while under* these delusions, religion will tend in any way to remove the malady. The homœopathists indeed will chuckle at the idea, for it tallies exactly with their doctrine—"similia similibus curantur." If religion, or rather superstitious fanaticism in allopathic doses, causes insanity, why should not the same, in homœopathic doses, cure the disease? But these knotty points we leave to those who have the management of the insane, who can best test the efficacy or prejudice of the system of religious instruction. Dr. Millingen, who lately had the superintendence of the Middlesex Lunatic Asylum, does not give a very favourable opinion of the system under consideration.

"Nothing," says he, "can be more absurd than the assertion of the great benefit that arises from the patients being obliged to attend divine worship. The apparent tranquillity and attention with which they seem to listen to the chaplain's exhortations, are purely mechanical. A lunatic seemed much affected at a sermon, and even shed tears with seeming contrition; the subject of the discourse was the Trinity. When questioned on the homily which had thus affected him, he said it was one of the most beautiful sermons he had ever heard—all about the Emperor of Russia and the King of Prussia! Were it not for the moral discipline enforced in these asylums, and the presence of the keepers, their congregations would very frequently exhibit anything but a devotional appearance. During fourteen months' superintendence of Hanwell, out of upwards of one thousand patients, I had only four who were fit to receive religious consolation in sickness, and on the bed of death." 4.

Dr. Brown appears to be of the same opinion.

"In the employment of such an agent (religion) great difficulties occur—so great, indeed, as to discourage the most zealous of its advocates. These consist in determining the modes in which the effect may be best obtained. If its doctrines are taught to weak or perverted intellects, they may add to the confusion already existing; if its influences are brought prominently forward, they are apt to mingle with superstitious fears and delusions; if its duties alone are commented on, the doubting and ignorant may be left unsatisfied; if preaching is the vehicle, the attention may be fatigued and exhausted; if prayer, the sentiments may be strongly affected. These suppositions are all obviously founded upon the injudicious use of such an agent." 4.

We are aware, however, that it is just as useless to urge these objections to the enthusiasts in religion, as to talk of the danger of cultivating farms and extinguishing slavery on the deadly banks of the Niger, to Fowell Buxton, or the weeping widows and melting maidens of Exeter Hall.

We shall only glance at another modern mania—the entire abolition of all personal restraint in cases even of the most violent insanity. Like the Niger question, a storm of abuse, and of popular indignation is let loose on all those who even entertain a doubt on the subject of this new species of emancipation. It is in vain that we repudiate personal restraint, except where the lives of the sane and the insane are put in jeopardy by the loss of reason and the fury of animal passion. No restraint is ever to be employed! Yet the sentiments of some men, immortalized by the justness of their observations, the soundness of their views, and the humanity of their dispositions, might weigh in the scale against this modern morbid sensibility to the horrors of a muff or a strait-waistcoat. The amiable and experienced Pinel relates the following interesting case.

"A gentleman, the father of a respectable family, lost his property by the revolution, and with it all his resources. His calamities soon reduced him to a state of insanity. He was treated by the usual routine of baths, blood-letting, and coercion. The symptoms, far from yielding to this treatment, gained ground, and he was sent to Bicêtre as an incurable maniac. The governor, without attending to the unfavourable report which was given of him upon his admission, left him a little to himself, in order to make the requisite observations upon the nature of his hallucination. Never did a maniac give greater scope to his extravagance. His pride was incompressible, and his pomposity most laughably ridiculous. To strut about in the character of the prophet Mahomet, whom he believed himself to be, was his great delight. He attacked and struck at everybody that he met with in his walks, and commanded their instant prostration and homage. He spent the best part of the day in pronouncing sentences of proscription and death upon different persons, especially the servants and keepers who waited upon him. He even despised the authority of the governor. One day his wife, bathed in tears, came to see him. He was violently enraged against her, and would probably have murdered her, had timely assistance not gone to her relief. What could mildness and remonstrance do for a maniac, who regarded other men as particles of dust? He was desired to be peaceable and quiet. Upon his disobedience, he was ordered to be put into the strait-waistcoat, and to be confined in his cell for an hour, in order to make him feel his dependence. Soon after his detention, the governor paid him a visit, spoke to him in a friendly tone, mildly reproved him for his disobedience, and expressed his regret that he had been compelled to treat him with any degree of severity. His maniacal violence returned again the next day. The same means of coercion were repeated. He promised to conduct himself more peaceably; but he relapsed again a third time. He was then confined for a whole day together. On the day following he was remarkably calm and moderate. But another explosion of his proud and turbulent disposition made the governor feel the necessity of impressing this maniac with a deep and durable conviction of his dependence. For that purpose he ordered him to immediate confinement, which he declared should likewise be perpetual, pronounced this ultimate determination with great emphasis, and solemnly assured him, that, for the future, he would be inexorable. Two days after, as the governor was going his round, our prisoner very submissively petitioned for his release. His repeated and earnest solicitations were treated with levity and derision. But, in consequence of a concerted plan between the governor and his lady, he again obtained his liberty on the third day after his confinement. It was granted him on his expressly engaging to the governess, who was the ostensible means of his enlargement, to restrain his passions, and by that means to screen her from the displeasure of her husband for an act of unseasonable kindness. After this, our lunatic was calm for several days, and in his moments of excitement, when he could with difficulty suppress his maniacal propensities, a single look from the governess was sufficient to bring him to his recollection. When thus informed of impropriety in his language or conduct, he hastened to his own apartment to reinforce his resolution, lest he might draw upon his benefactress the displeasure of the governor, and incur, for himself, the punishment from which he had but just escaped. These internal struggles between the influence of his maniacal propensities and the dread of perpetual confinement, habituated him to subdue his passions, and to regulate his conduct by foresight and reflection. He was not insensible to the obligations which he owed to the worthy managers of the institution, and he was soon disposed to treat the governor, whose authority he had so lately derided, with profound esteem and attachment. His insane propensities and recollections gradually, and at length entirely, disappeared. In six months he was completely restored. This very respectable gentleman is now indefatigably engaged in the recovery of his injured fortune." 79.

We shall only add the following sentiments of Mr. Bingham himself, whom we know to be one of the most tender-hearted and humane mortals existing in this world.

"There exists in the public mind, at this time, a strong feeling against all kinds of personal restraint in lunatic establishments, and some practitioners have proposed to banish it entirely from them. I have no doubt it is *practicable* to do so by multiplying keepers and nurses, to supply by manual coercion and watching the place of those simple contrivances which are now in use, and by providing rooms, the sides and floors of which are so padded, that the most unruly patient could not injure himself by falling against them. But whether any benefit would accrue to the patient by such a change of system, is a point that must not be lost sight of. I am acquainted with the result, in the hands of some who have tried it, and it is by no means encouraging. The patients suffer *much more*, I am informed, by resisting the combined attempts of several persons to coerce them, than they would by the preventive efficacy of well-contrived muffs, sleeves, and other means for keeping them out of mischief." 81.

We are entirely of Mr. B.'s opinion on this point; but granting, for the sake of argument, that in some public asyla, as Hanwell for instance, such a system of entire non-restraint could be put into execution by legions of keepers and other auxiliaries, how could it be effected in private houses, consistent with any reasonable degree of economy? Then the numerous cases of head-long propensity to suicide, to masturbation, and twenty other practices subversive of health and even life—how are they to be attended to? But, as we said before, it is of no use to argue with enthusiasts. The only feasible plan that we can conceive, is, to have a mesmeric professor—an animal magnetiser in each asylum; whose business it may be to throw all turbulent spirits into profound sleep every evening, and awake them with his fugle-motions or *reveillez* at sunrise.

